# Use of Frame Running for Adolescent Athletes With Movement Challenges: Study of Feasibility to Support Health and Participation

**DOI:** 10.3389/fspor.2022.830492

**Published:** 2022-03-09

**Authors:** Theresa Sukal-Moulton, Tara Egan, Larke Johnson, Crystal Lein, Deborah Gaebler-Spira

**Affiliations:** ^1^Department of Physical Therapy and Human Movement Sciences, Feinberg School of Medicine, Northwestern University, Chicago, IL, United States; ^2^Department of Pediatrics, Feinberg School of Medicine, Northwestern University, Chicago, IL, United States; ^3^Office of Diverse Learner Supports and Services, Chicago Public Schools, Chicago, IL, United States; ^4^Chicago Run, Chicago, IL, United States; ^5^Shirley Ryan AbilityLab, Chicago, IL, United States; ^6^Department of Physical Medicine and Rehabilitation, Feinberg School of Medicine, Northwestern University, Chicago, IL, United States; ^7^Lurie Children's Hospital, Chicago, IL, United States

**Keywords:** participation, childhood disability, fitness, frame running, sport based youth development

## Abstract

Children and adolescents with movement challenges have lower instances of physical activity and longer time spent in sedentary behaviors compared to children with typical development. The purpose of this study was to investigate the feasibility of a sport-based youth development running program modified for accessibility using a running frame and to evaluate initial evidence for its efficacy on endurance and functional strength. We completed four 8-week seasons (2–3 times per week) in a combination of 3 different formats by season: online remote (winter and spring), in person in a community park (winter, spring, and summer), and in person in an afterschool setting (autumn). Participants included 13 athletes (average age 14.46 years, range 8–18 years, 4 females), who collectively completed 22 season blocks. Diagnoses included cerebral palsy (*n* = 10), arthrogryposis (*n* = 1), Dandy-Walker malformation (*n* = 1), and transverse myelitis (*n* = 1). In all settings, participants engaged in activities of social emotional learning, cardiovascular endurance, and muscle strengthening in a progressive manner. We found that each season format was feasible to administer with high attendance rates (76–97%) and positive qualitative feedback from athletes. In addition, promising average improvements in motor performance across a season (6 min frame running test, 170 m; timed up and go test, 8.44 s; five times sit to stand, 14.1 s; and Goal Attainment Scale, *t* = 65.01) were identified in the pilot data of this non-randomized cohort. Training in any of the proposed settings with an overall goal of completing a community race in a running frame is feasible and warrants further study.

## Introduction

There are widely established benefits to exercise in children and adolescents including cardiovascular and musculoskeletal as well as academic and mental health benefits (Herting and Chu, 2017) for children of all ability levels. These benefits are also clear in regards to physical activity for persons with disabilities (Carroll et al., 2014) and the American Academy of Pediatrics has recently released a policy related to this (Carbone et al., 2021) where they emphasize the importance of exercise for all patients, regardless of ability level. For conditions such as cerebral palsy (CP), an initial and non-progressive injury to the developing brain may still cause secondary cardiovascular consequences due to lack of movement and physical activity. These co-occurring conditions occur in adulthood at a higher rate and earlier onset than the population of adults without CP (Ryan et al., 2019; Peterson et al., 2020; Thorpe et al., 2021). Although data are limited, there is reason to believe that a lack of moderate to vigorous physical activity and high levels of sedentary time (Verschuren et al., 2016) during the course of a day may be at least partially responsible for these conditions seen in adulthood.

CP has an emerging field of evidence associated with activity and participation, but children with other diagnoses that impact movement would also likely benefit from increased access to participation in activities that encourage movement and elevate heart rate. However, children with disabilities and movement challenges have difficulty accessing programs targeted at improving physical fitness (Martin Ginis et al., 2016). In addition, they have 38% higher rates of obesity in childhood (Grondhuis and Aman, 2014), which may be linked to a greater risk of cardiovascular comorbidities (Peterson et al., 2015; Edwards, 2018), pain (Jahnsen et al., 2004), and fatigue (Malone and Vogtle, 2010) in adulthood.

Youth are significantly influenced by environmental factors including relationships with peers and mentors. Sport-based youth development is a strategy that aims to promote healthy behaviors in conjunction with social confidence (Curran and Wexler, 2017) through athletic games, team building, and emotional learning opportunities. Running is an appealing option for sport-based youth development as it requires virtually no equipment, can have both individual and team aspects, and accommodate children of various skill levels. Importantly, it is also an activity that can be maintained over a lifetime, particularly if a strong foundation and interest is built early in life.

The translation of a skill such as running can be difficult for those with motor challenges especially when they typically use more restrictive orthoses or equipment for ambulation. With community locations such as fitness centers often less accessible (Martin Ginis et al., 2016), fitness opportunities need to be sought elsewhere.

**Figure 1:**
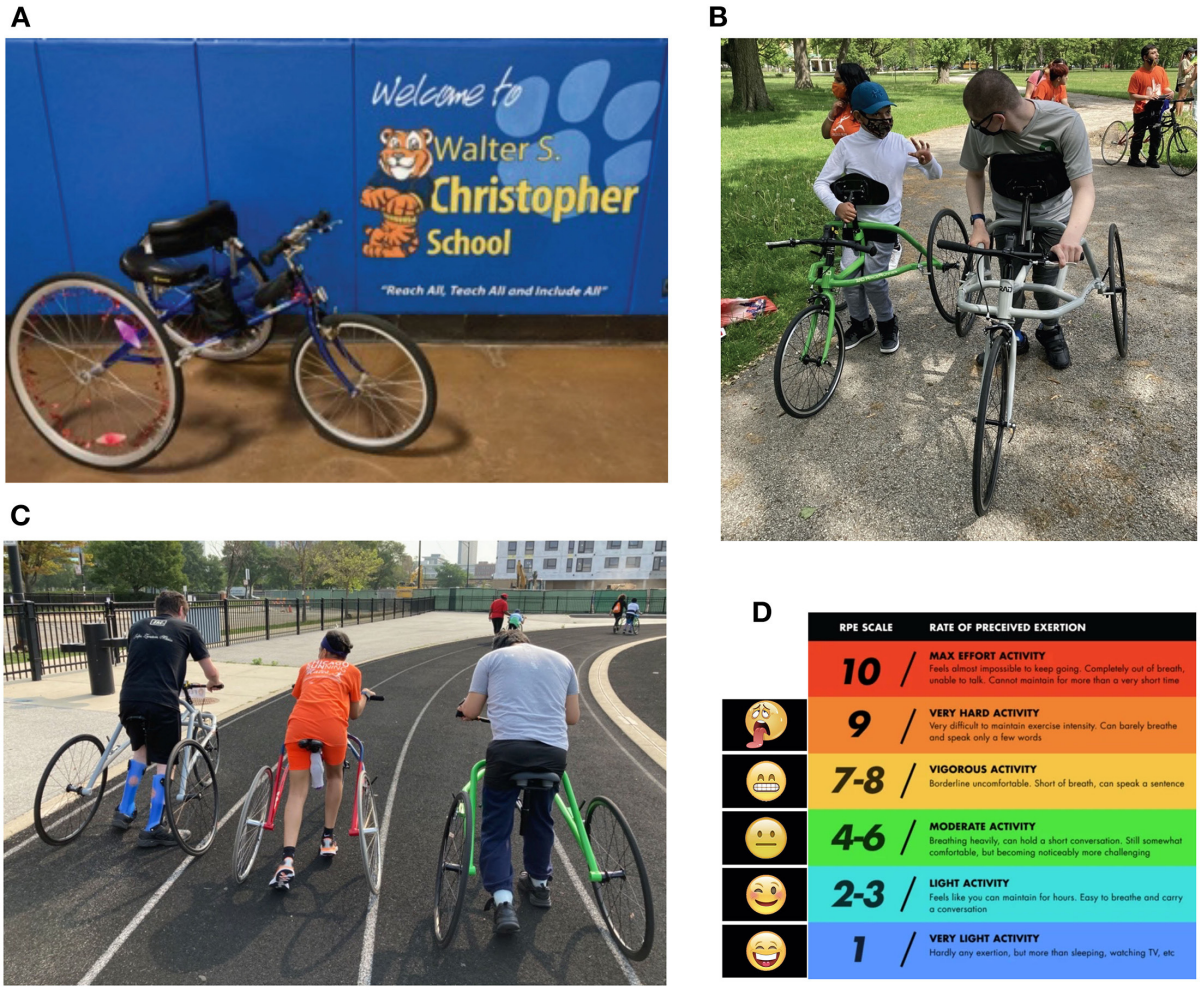
**(A)** Running frame example. There are 3 wheels, a seat (styles of seat vary in shape and dimension), and a chest plate for additional trunk support. The front wheel is held steady with the use of a damper. Bicycle style breaks on the front wheel allow athletes to stop and wheel locks on one or both back wheels secure the frame for mounting and dismounting. The model shown here is by Petra (Peterson et al., 2015). **(B)** Front view of athletes using a running frame during a social and emotional learning (SEL) teamwork activity at practice. Both frames are by RAD (Black, 2021). **(C)** Back view of running frames in use during an endurance portion of practice by three athletes on a track to show relative width of the back wheels; they are wide enough for stability and fit through ADA-compliant doorways. The frames on right and left are RAD (Black, 2021), and middle is Eagle Sportschairs (Ewing, 2021). **(D)** Rate of perceived exertion (RPE) scale image used by athletes to report their perceived level of effort. This image was shown on shared screen during virtual sessions, and on a laminated index card during in-person sessions.

A running frame is a 3-wheeled device with a seat and frame and no pedals (Figures 1A–C). It was created to allow for participation in running for people who have disabilities that affect their movement. The first running frame (initially called a RaceRunner) was constructed in Denmark in 1991, and they have extended their reach across Europe. There is an international competitive association for frame running (http://www.racerunning.org). Although limited, the data for using these devices for therapeutic indications are encouraging. Preliminary evidence is positive for changes in bone mineral density (Bryant et al., 2015; Van Schie et al., 2016) and muscle thickness (Hjalmarsson et al., 2020), but formal evaluation of running programs performed using these tools is limited, particularly in the American context.

The purpose of this study was to pilot an intervention of a sport-based youth development program modified for accessibility for children and adolescents with movement challenges, with the goal of community-based running participation using running frames. In addition, we sought to investigate the feasibility of offering such a program using a variety of formats, and to evaluate initial evidence for its efficacy on endurance and functional strength.

## Materials and Methods

This non-registered feasibility study had a non-randomized design with a sample of convenience that occurred in the City of Chicago, Illinois, USA. Athletes were invited to participate if they were between 6 and 18 years of age (covering both childhood and adolescent phases of development), if they had a diagnosis that made running difficult for them, and no medical concerns that made participation unsafe for them (for e.g., poorly controlled seizures, bone fracture risk, or orthopedic surgery within the previous year). Potential candidates were selected from the caseloads of the physical therapists in the trial; parents were sent details about the team, goals of the program, and logistic details. Parents and participants were given the opportunity to ask questions, and they completed an informed consent and assent, respectively. In addition to the informed consent document, parents/guardians completed the Chicago Run participation waiver. They also needed access to a reliable internet connection for remote sessions; there were several government programs in the city at the time to support students learning remotely, which reduced the barrier of this requirement. At the time of enrollment, the interest/need for transportation was assessed and athletes were provided or offered a para-transit bus to attend practice. To assess general community resources, participant zip code was found on the US Census American Community Survey (US Government, 2021), and the number of unrelated adults living below the poverty line within participant zip codes was extracted as a proxy for relative level of resources available within a local community.

Our study was set to begin in the winter of 2020. Consistent with previous studies by other investigators (Hjalmarsson et al., 2020), the initial strategy was to have all sessions conducted in person after school. However, fully remote school education for much of the 2020–2021 school year meant that an alternative strategy was required (Egan et al., 2021). Guided by the local gathering restrictions in place at the time, we evaluated whether each season should include virtual components or be entirely in person. The first season began in January of 2021, and was entirely virtual until the weather was suitable for outdoor practices to commence in city parks in late February of 2021. By summer, when vaccination rates were improving and cases of COVID were decreasing locally, we hosted the practices entirely in person outdoors with masking and distancing procedures in place. In fall, school returned for in person learning and we launched the after-school format. The school location was also safe and felt familiar to the athletes where they were co-located with students who do not have disabilities. The summary of each season can be found in Table 1.

**Table 1:** Example training session schedule.

	**Online**	**In person**
Social emotional learning (10–15 min)	Check in with how everyone is feeling Two truths and a lie: athletes take turn sharing 3 statements, 2 of them are truthful and one is made up. The team attempts to distinguish the untrue one	Check in with how everyone is feeling “Sole mates” activity: athletes move their running frames to pair up based on a physical similarity called by coach (example, both wearing same color shoes), and then share with each other something about themselves (example, what do you like to eat for breakfast?)
Strength focused activities (10–15 min)	Sit to stands or squats (chosen by coach or athlete based on ability/safety): 3 × 15 reps Seated leg extensions using a resistance band: 3 × 15 reps	Frame running with resistance provided by coach using resistance band: 4 × 100 m Hill navigation in running frame: 4 × 30 m
Endurance focused activities (20–30 min)	Circuit of the following exercises x2–3 min each (options chosen by coach or athlete based on ability/safety): - Step ups or marching in place - Seated punches with water bottles for resistance or jumping jacks - Side steps or jumping jacks - Running in place or speedbag punches	Pacing activity rotating between frame running for 1 min at slow speed (“turtle”), 3 min at medium speed (“dog”), 30 s at fast speed (“cheetah”), repeated 5 times, ending with 5 min on medium speed. Completed on a loop so athletes are near one another even though they may move at different speeds.

To support the in-person components of the training, running frames were fit individually for each athlete. There were a number of frames available [Petra (Hansen, 2021), Eagle (Ewing, 2021), and RAD (Black, 2021)], and the sizes were selected by approximate inseam length and lateral stability offered by the frame. The seat height and depth were adjusted for distance to the ground, and seat styles were chosen for comfort by each athlete. Handlebars were generally kept in stock condition (as seen in Figure 1), but the height/angle were occasionally adjusted to be closer to the participant using either the rotation ability of the device, or through the addition of a stem extender. Frames were stored for athletes between sessions.

Chicago Run is a non-profit organization that provides young people from Pre-K through high school with inclusive running and physical activity programs. Chicago Run's *Running Mates* program utilizes running and physical activity to improve the social and emotional wellbeing of young people in middle school and high school. This program provides an inclusive and non-competitive environment in which young people work toward completing a community race, while developing a positive self-image, promoting goal setting and building relationships with peers. It is highly tailored to the needs of the athletes and settings in which it is administered, but generally includes elements of team building/emotional growth, and building endurance for running activities. The Chicago Run curriculum was adapted for the online format and to accommodate running frames when in person.

Each practice was generally formatted to include a focused social emotional learning (SEL) element, activities aimed at cardiovascular fitness (to elevate heart rate), and strengthening activities. SEL activities were designed to help athletes learn more about themselves (such as goal setting or identifying their own strengths) and their teammates. Cardiovascular activities were graded to increase duration and intensity over time, aiming to meet at least 20 min of continual activity completed at a moderate to vigorous intensity level (Verschuren et al., 2016). Athletes were educated in the Rating of Perceived Exertion (RPE) scale (Fragala-Pinkham et al., 2015). This tool was used to promote body awareness and help monitor the intensity of activity. The scale was set between 1 and 10, with both numeric values and emoticon representations, shown in Figure 1D. The aim was to obtain the RPE two to three times during the cardiovascular endurance section of the practice, and once during or at the conclusion of strength training. Online training was completed at a set time during the day when coaches and athletes all joined a video conference together (Zoom). This format was conducive to many of the SEL activities as they involved speaking and sharing; some athletes preferred to use the chat box feature, while others preferred to use verbal participation. Examples of strength-based activities included sit to stands or squats, step ups to a small folding step provided to athletes, resisted lower extremity activities using a provided resistance band, and standing hip abduction exercises. We attempted floor-based activities for safety, but it was more challenging to coordinate the camera and appropriate spaces for these activities for athletes in their home environments. Cardiovascular endurance activities varied by athlete ability and safety. Some athletes completed standing activities such as repetitive step ups, side shuffles, or jogging in place, while others elevated heart rate using activities such as seated arm punches to various directions with or without water bottles for resistance or arm cycles at various speeds. As the season progressed, some athletes also completed full body activities such as jumping jacks. We utilized a demonstration of activities by coaches, teammates, or animated movies, and we often used a screen-share of a timer to show progression in the activity. When in person we met as a group at an outdoor community park or the indoors in school hallways or the gymnasium. Almost all endurance-based activities were completed on the running frame with an increasing time goal for movement (at any speed) each week. Strength activities in person were also completed using the running frames with a focus on high speed, using resistance (resistance bands applied by a coach to make it more challenging for the athlete to move forward), or incorporating hills or ramps. The SEL activities in person presented the opportunity for kinetic problem solving such as connecting partners by pool noodles to navigate an obstacle course as a team or playing a card game while collecting cards from different areas of the gym. An example of each session type is shown in Table 1. Four seasons were completed with 4–8 athletes participating each season. The format of each is specified in Table 2. For all seasons, athletes competed in a community run at or toward the end of the training. They could chose to complete either a 1-mile or 5-km distance with other runners in our local community at a city park usually in proximity to their neighborhood (Chicago Area Runners Association, 2021). This season-long goal is consistent with Chicago Run teams at other schools and sites that do not use running frames and was timed in conjunction with other Chicago Run teams as possible; family members of the athletes were invited to join them in the run. Head coaches in our program included 2 physical therapists (authors TS-M and TE), each with more than a decade of clinical experience in pediatrics and knowledge of a variety of equipment types and extensive experience in adjusting equipment and titrating exercise for individual needs. Neither has been certified in frame running coaching, but both obtained experience through research, consultation with manufacturers, discussions with local adaptive sports organizations, and trial and error. Assistant coaches varied by season, but included 1 physical therapist, and 2 paraprofessionals with experience in working with children with movement challenges in a school setting.

**Table 2:** Season formats.

	**Format**	**Coaching support**
Season 1: Winter (4 athletes)	4 weeks of 2 times per week online (45 min per session) remote 4 weeks of 2 times per week online (45 min per session) plus 1 × /week (60 min per session) in person	2 DPT head coaches Chicago Run program staff*
Season 2: Spring (8 athletes)	8 weeks of 2 times per week online (45 min per session) plus 1 × /week (60 min per session) in person at pubic park location	2 DPT head coaches 1 Chicago Run Junior Coach Chicago Run program staff* Intermittent parent assistance
Season 3: Summer (6 athletes)	8 weeks of 2 times per week in person (60–90 min per session) at public park location	2 DPT head coaches 1 Chicago Run Junior Coach 1 DPT, 2 paraprofessional assistant coaches Chicago Run program staff*
Season 4: Autumn (4 athletes)	8 weeks of 2 times per week in person (90 min per session) after school in hallways and gymnasium	2 DPT head coaches 1 DPT, 2 paraprofessional assistant coaches Chicago Run program staff*

*Consistent with other Chicago Run program sites, staff provided curriculum content, access to resources, and attended practice ~1 session per week.

We used the 500 m distance of the Functional Mobility Scale (FMS-500) (Graham et al., 2004; Harvey et al., 2010) to describe how the participants typically maneuvered around their community. Attendance rate percentage was calculated for each athlete each season as the number of sessions attended divided by the number of sessions offered that season. The following outcome measures were collected at the beginning and end of each season: 6 min RaceRunning test (Bolster et al., 2017), or 6 min frame running test (6MFRT), as a measure of endurance while in the running frame; Timed Up and Go (TUG) test (Carey et al., 2016) as a measure of functional mobility; and five times sit to stand test (5xSTS) (Kumban et al., 2013) as a measure of functional strength. Each participant contributed to their own goals using the Goal Attainment Scale (Steenbeek et al., 2007), a tool designed to standardize the setting and scaling of goals in a way that allows for both improvement and regression as a result of intervention. GAS has been used to show meaningful change across a range of diagnoses and intervention types in pediatric rehabilitation (Harpster et al., 2019). Goals were collaboratively identified starting with what was important to the participant (“I want to be go from the 1 mile distance to the 5K distance in the race, but it is a big jump and I am not sure I can do it”). The investigator and participant worked together to determine current level (“I can finish the mile like last season,” GAS = −1), regression (“I have to stop before completing the mile,” GAS = −2), expected level (“I will complete 2 miles,” GAS = 0), greater than expected (“I will complete 2.5 miles,” GAS = 1), and much greater than expected (“I will complete the full 5K,” GAS = 2). All athletes were encouraged to have at least 1 participation-based goal in a process consistent with the framework proposed by Krasny-Pacini et al. (2016). The results were converted to a T-score for overall goal attainment within a season for comparison across participants. Non-blinded physical therapists completed the assessments using standardized protocols for each. In addition, we asked for feedback from participants using a semi-structured interview about the season and their self-perception and enjoyment as an athlete (starting with the broad question of “what are your thoughts about this program?” with follow-up questions based on their responses). We asked other stakeholders (parents and teachers) if they noted any differences during the athlete's participation in the program. Data collected from interviews through written notes, audio recordings and field notes from throughout the season were evaluated by authors using thematic analysis. The study was approved by the institutional review board of Northwestern University and the research review board of Chicago Public Schools. Participants were able to withdraw from the study at any time and still participate in the curriculum as long as they maintained the waiver to participate with Chicago Run.

Study data were collected and managed using REDCap (Research Electronic Data Capture) tools hosted at Northwestern University (Harris et al., 2009, 2019). To evaluate the feasibility of the program we summarized the percentage of sessions attended, comments made by parents, athletes and coaches, and the pilot results of outcome measure differences. Descriptive statistics were used to evaluate differences between pre and post outcome measures, as well as comparison to thresholds of detectable or meaningful difference. The reported RPE and attendance each day were evaluated using a Kruskal–Wallis test to examine the impact of location (3 levels: online, park, and school). A *p* < 0.05 was considered to be significant, and software used was IBM SPSS statistics, Version 27.

## Results

A total of 13 athletes participated in the program, with 22 season blocks in total. Some athletes participated in a single season (*n* = 8), and others appeared in 2 seasons (*n* = 1) or 3 seasons (*n* = 4). Diagnoses included cerebral palsy (CP, *n* = 10), arthrogryposis (*n* = 1), Dandy Walker malformation (*n* = 1), and transverse myelitis (*n* = 1). The FMS-500 of participants included those who walked without assistive devices, but had difficulty in crowds (FMS-500 = 5, *n* = 4), one who used bilateral forearm crutches (FMS-500 = 3), one who used a reverse walker (FMS-500 = 2), and several who used a manual or power-assist wheelchair (FMS-500 = 5, *n* = 6). Average age of the participants was 14.46 years on the first day of their participation, and ranged between 8 and 18 years. There were 4 athletes with biological sex of female, and 9 who were male. The zip codes where athletes lived had an average of 26.4% of adults with an annual income below the poverty line (range 17.4–34.7%). Two athletes used the bus transportation in winter, spring, and summer; and 1 athlete used the bus in the summer only.

Starting in the spring season one of the athletes served as a Junior Coach for the team (author LJ), taking a role in planning ~1 activity per session, encouraging teammates, and leading through example within the group. In addition, we had one participant that was the sibling of an athlete and did not have a disability. She initially joined as a volunteer, and eventually was engaging in most aspects of team activities. Consistent with our plans, there was always at least one head coach/licensed physical therapist at practice, with additional support as shown in Table 1.

Our overall attendance average across all seasons and participants was 79% of offered sessions. Two athletes (15% of cohort) chose to stop attending sessions early in the program due to scheduling constraints and not enjoying the program enough to continue. Both athletes who stopped attending were diagnosed with CP (ages 15 and 17 years), and had an FMS-500 score of 5. To assess feasibility in terms of barriers to ongoing participation we removed them from subsequent descriptive results. The remaining 11 participants demonstrated an 86% attendance rate across all seasons combined. In the winter season, we had an average attendance rate of 97%; spring season was 87%; summer season was 82%; and autumn season was 78%. Among the athletes that were provided transportation (*n* = 7 season blocks) the rate was 89%. Attendance divided by location showed 83% of online sessions were attended, 85% of the sessions offered at a community park, and 78% of those hosted after school. The Kruskal–Wallis test did not reveal differences of attendance based on practice locations (*H* = 1.42, *p* = 0.49). Reasons for non-attendance included vacations during times that school was not in session, COVID-19 exposures or illness, death in the family, and medical procedures/appointments.

**Figure 2:**
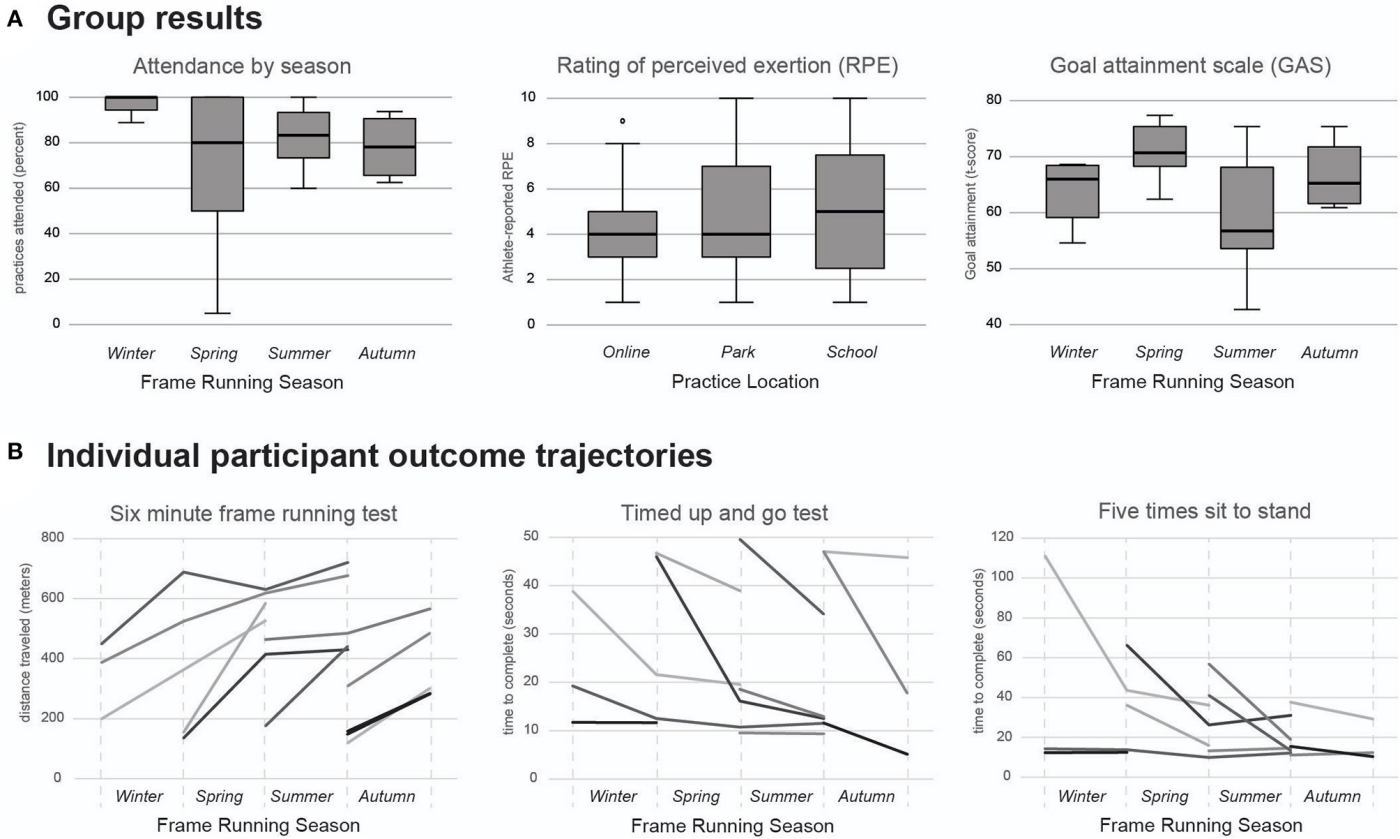
**(A)** Group summary box and whisker plots. The practice attendance percentage is calculated by dividing the number of practices attended by the total number for a season. This panel includes all 8 athletes that began the spring season. There was a significant main effect of location found in the Kruskal–Wallis test of RPE (*H* = 42.38, *p* < 0.001), where park and school were both significantly higher than online; the median (shown in thick black in plot) of home was four, park was four, and school was five. GAS *t*-scores are calculated to combine each athlete's overall goal attainment within a season; with coaching and support from researchers, athletes set 2–4 goals each season. **(B)** Individual data trajectories shown for performance-based outcome measures. Each line represents a different participant. For reference, the normative TUG for 5–13 year old youth ranges from 6.20 to 7.12 s (Itzkowitz et al., 2016), and the 5xSTS for 14–19 year old youth is 6.5 s, with a standard deviation of 1.2 s (Bohannon et al., 2010).

Our secondary goal was to evaluate the initial efficacy of the intervention. We did receive reports of minor muscle soreness or fatigue after practice (<2 reports per week across all athletes), particularly early in the season. Ongoing tweaks to running frame settings were made as needed to improve comfort or running form. There were no injuries requiring physician follow-up, and no complaints outside of the musculoskeletal system. Athletes reported RPE (*n* = 412 total data points) at a range of levels (from 1 to 10) that were generally reflective of outward signs of exertion such as heavier breathing or sweating (mean RPE = 4.52, standard deviation, SD = 2.36; median RPE = 4.00, interquartile range = 3). The Kruskal-Wallis test revealed a main effect of location (*H* = 42.38, *p* < 0.001). Athletes reported significantly higher RPE in person (either park or school) compared *post-hoc* to RPE reported online (mean, SD for school = 5.36, 3.11; park = 4.81, 2.38; online = 4.03, 1.87), seen in Figure 2A. There was inconsistent collection of the RPE in certain seasons, especially when athletes were further spread apart in distance.

With respect to outcome measures, we did have some gaps in research-quality data; longitudinal data is shown in Figure 2. Considering the data available, the 6 min frame running test improved by 170.4 m over a season (*n* = 16 pre-posttest pairs, SD = 135.6 m). Using the most conservative minimal detectable change (MDC) according to Bolster et al. (2017) of 172 m, 6 athletes surpassed the threshold of change. The TUG improved by 8.44 s (*n* = 15 pre-post-test pairs, SD = 10.07 s), with 8 athletes surpassing the minimal clinically important difference (MCID) of 5.31 s (Carey et al., 2016) during a single season. The 5xSTS improved by an average 14.1 s (*n* = 15 pre-post-test pairs, SD = 20.74 s), with 6 athletes surpassing the MDC of 0.11 reps/s (Wang et al., 2012) in a single season. The GAS T-score average was 65.01 (*n* = 20 season end assessments, SD = 8.92), with 14 athletes achieving a “successful” season using the criteria of Desloovere et al. (2012) (*T*-score > 60). We completed community-based runs at distances of 1 mile (*n* = 20; completed at Garfield Park, Humboldt Park, Jackson Park, Columbus Park and River Park in Chicago, IL; the number of participants in each race was limited to 150 registered runners due to COVID-19 policies of Chicago Area Runners Association) and 5 km (*n* = 3; completed at Humboldt Park with ~75 runners and the Great Lakes Adaptive Sports Association Twilight 5K with ~150 runners and para-athletes), as well as an in-school celebration run completed at multiple distances (0.6, 1, 1.2, and 1.7 miles, *n* = 1 athlete running each distance). At every race, each athlete had at least 1 family member attend to cheer for them or to run with them. One athlete in the winter season decided to not participate in the run due to concerns about COVID-19 precautions and potential exposures, but was able to complete the 1-mile distance during a practice at the end of the season.

Qualitatively, we completed 21 interviews (18 for athletes at the end of their season, 1 paraprofessional, and 2 teachers), and 6 coaches/Chicago Run staff contributed to field notes. We found a few themes in our data. The most common sentiment was an enjoyment of physical activity and happiness to be moving and active after the time spent in isolation during COVID-related quarantine. Within this overall theme, athletes noted that they liked using the running frames to move, and also enjoyed interacting with peers in a team setting. Athletes expressed opinions such as “exercising is winning,” and “it is been a while since I have been outside and it was so nice to see everyone.” One athlete's paraprofessional noted that he was more positive throughout the day during the running program, and that he expressed sadness about it coming to an end. In addition, our field notes detailed several instances of informal leadership and strong team behaviors, including verbal encouragement for elevated performance between athletes, providing clear instructions to one another during a tethered obstacle course, and a younger athlete sharing with coaches that they looked up to one of the older athletes and their abilities.

A second theme identified was pride for goal accomplishment and noticing results related to effort put into the running program. Specifically, there was a lot of positive expressions related to the community run. One participant was asked what the best part of their summer had been on the day of their community run, and their response was “today.” Another athlete noted, “We are a family of runners now,” in reference to the participant's father and mother being runners who also completed community races. In addition to goals documented with GAS, some athletes said they could move more easily (stairs, walking were examples shared by athletes). Following the autumn season, one teacher shared her impression that an athlete in her class was sitting with better posture in his wheelchair throughout the day while participating in the program.

A third theme identified in field notes and interviews was the impact of the community environment on the athletes' efforts and pride in participating. In winter, spring and summer, our celebration race was held at a community race where we had the opportunity to raise awareness about athletes with movement disabilities within our local community. Fellow runners and spectators demonstrated support and encouragement that athletes said increased their enjoyment and pride of accomplishment. Our autumn season celebration race was held in the school, and was attended by parents of the athletes as well as other students attending a different afterschool program. Notably, we observed non-disabled peers of the athletes spontaneously voiced words of support, made signs of encouragement, and cheered for the duration of the race, with some spectators noting, “I am so proud of them.”

Finally, there was a theme related to a lack of reliable access to fitness outside of the program. A minority of participants discussed other structured activities they were involved in, and many shared they were otherwise engaged in predominantly sedentary activities during their day. When accessing our program, barriers to attendance included technology difficulties (online sessions), transportation (in person), or competing events (both formats). Those that used the provided transportation (*n* = 2 in winter and spring and *n* = 3 in summer) stated they would have been otherwise unable to participate due to limitations in transportation access. Everyone who completed a season said they would sign up for another one.

We did not find any consistent themes related to location of practices, and no clear preference between session type for athletes experiencing both online and in person; both were deemed acceptable and generally easy to attend. However it was noted that being in person was “not the same as a computer or iPad.” In addition, we did not complete interviews with the athletes who decided after a limited number of sessions that they were not interested in continuing. One cited that they felt the program was too juvenile in the activities and the other was hesitant to try the running frames at enrollment and did not find them comfortable.

## Discussion

This preliminary study provides evidence for the feasibility of the use sport-based youth development and running frames to support fitness and participation goals for athletes who have movement and/or balance challenges. We found a number of useful lessons for future seasons by trialing a variety of formats, providing for flexibility and opportunities as the uncertainty of the pandemic continues.

The program in this study addressed many of the common barriers and facilitators noted by the American Academy of Pediatrics (Carbone et al., 2021), including access to transportation, supervision, a focus on social learning, physical literacy, and individual goals at activity plans. It also is consistent with the recommendations of a recent international clinical practice guide (Jackman et al., 2021) and there is a demonstrated interest for programs aimed at participation-based exercise for youth with disabilities (Shields et al., 2018). Our cohort confirmed that they liked to exercise, and needed appropriate outlets to do so. Supportive relationships and service availability are key factors in community participation (Willis et al., 2017). The use of online programming was safe and well-attended, including by families who were extra cautious about the spread of COVID-19. An advantage of the virtual format was the use of features such as screen sharing, chat box, and polls to facilitate activities and communication. It was feasible for one coach to lead several athletes. In addition, we were not limited by the physical distances of where athletes and coaches were located and athletes could participate from their own home, which lead to high convenience as has been shown in other studies (Rowland et al., 2016; Astley et al., 2021; Calcaterra et al., 2021; Weiss et al., 2021). However, it was difficult at times to fully engage remote athletes in a way that was both safe and challenging without anyone close by to supervise for their specific movement challenges.

While in person, athletes appeared more likely to fully engage in an activity, able to push themselves a further with guidance and supervision of coaches and teammates and perhaps through the use of the running frame to support movement, as evidenced by the RPE differences. In-person practices were overall safe, as some musculoskeletal discomfort was an anticipated potential side effect of engaging in a new activity. These reports were few, minor, and diminished as the season progressed, indicating a training effect and/or finding optimally comfortable settings. Being outdoors was a further advantage because it offered more safety related to spread of COVID-19 in combination with other precautions such as cleaning protocols and temperature monitoring. The less desirable aspects of in-person included potential impact of the weather and added cost for additional coaches and transportation.

The afterschool format was more like programming aimed at children without disabilities and took advantage of multiple athletes already being in one location. Joint activities where students with and without disabilities work together have a positive impact on the attitude toward students with disabilities, highlighting the advantage of peer interactions and support (Alnahdi et al., 2021). This demonstrates the value and importance of active participation by all students in their community and peer group. It was anticipated that attendance would be higher given the convenience of programming at school, but we found there were higher rates of schedule conflicts during this season. Missed practices appeared to be at least partially attributable to pandemic related precautions and circumstances such as exposure to someone infected with COVID-19. In addition, following a pause in certain medical care for the year prior (Sutter et al., 2021) families may have been catching up on medical appointments for routine care such as eye exams or physiatry visits. Children with movement challenges have higher healthcare needs in general, and future expectations for attendance may need to take some of these factors into consideration. Like other programs in a school setting (Cleary et al., 2017), our data from the autumn season suggest improvements in endurance (6MFRT).

Each of the three modes of intervention was found to be feasible and with different benefits and challenges. In combination with in-person programming, the use of remote practices ongoing would be a feasible way to increase dosage without the added cost of transportation or time constraint for travel. When in person, we found that athletes engaged in higher perceived intensity of exercise, potentially due to the combination of balance support and freedom of movement provided by the running frame. This mode of delivery also provided for specificity of practice for the team goal of community race participation. The option for transportation was critical for equitable access to the program by all who could benefit (Aviram et al., 2021). The locations where most of our participants live and attend school are within 26 areas of high economic hardship in the city of Chicago and in areas of low childhood opportunity (Health, 2021). This highlights a need for additional supports to ensure equitable access to physical activity.

Provision of a program such as the one introduced in this study fills an important gap in options for physical activity. A study of almost 2,000 children with CP in Sweden (Degerstedt et al., 2021) found that while most (87%) participated in physical education class at school, barely half (58%) had physical leisure activity. International guidelines highlight the importance of exercise and muscle strengthening for CP (Damiano et al., 2021). In addition to a focus on reducing sedentary behaviors and increasing physical activity in general, Verschuren et al. recommend that cardiorespiratory exercise be completed 2–3 times per week for a minimum of 20 min, at more that 60% peak heart rate, or more than 40% of the heart rate reserve (Verschuren et al., 2016). We achieved the time target in all seasons in the current study, and based on the RPE and observation, the intensity level was at least moderate, although this will require future study. We are exploring ways to record RPE and heart rate more consistently, including with athletes who have challenges in communication or for all athletes as they are still learning to connect physiologic signals with reported numbers. Although 8 successive weeks of training are recommended, a lifestyle practice is most likely to result in optimal health benefits (Verschuren et al., 2016). In our athletes who completed more than one season, we also found continued improvements in their running times and outcome measures. When administered in higher dosages, other running interventions have shown improved running abilities, particularly those with CP who could walk unaided at the onset of the program (Gibson et al., 2018). Programs shaped in an individual's personal goals added to their enjoyment of the program (Kahlon et al., 2019). Consistent with these studies, we found changes in physical performance for athletes participating in our frame running focused fitness group.

We also found strong community support for the athletes as well as strong parental support previously shown to be important in youth physical activity (Ku and Rhodes, 2020). In mainstream media, there is emerging coverage of talents and abilities that individuals with mobility impairments possess. For examples, the professional sponsorship of Justin Gallegos (Cash, 2018), coverage of Paralympic events and documentaries (Ferrara et al., 2015; Coates and Vickerman, 2016) of multi-medal athletes such as Tatyana McFadden (Bonhôte and Ettedgui, 2020; Committee, 2021b) and Ryley Batt (Committee, 2021a), and the inclusion of dancers with disabilities in professional performances (Caldwell, 2019; Chicago, 2021; Dubon et al., 2021) are becoming more common. The positive messaging associated with this type of accomplishment lays the foundation for young children with similar physical challenges to envision themselves also achieving fitness and athletic goals. Similar to the qualitative experience of adults participating in adaptive sports (Lape et al., 2018), the cohort of athletes in our study already had general social interactions during practices at baseline, but continued to grow through the use of social-emotional learning and goal acquisition. This is an area of opportunity to explore more deeply in future work.

Although our study was not adequately powered to evaluate outcomes in the body structure and function domain, pilot findings in the activity domain (TUG, 5xSTS, 6MFRT) are encouraging and warrant further investigations. The study does demonstrate that several modes of intervention administration are feasible and can be tailored to individuals, teams, or communities for maximal impact. If targeted and engaging intervention makes an impact during adolescence, there's promise of shifting behaviors toward more physical activity and fitness across the lifespan.

## Data Availability Statement

The datasets presented in this article are not readily available because the data set is limited due to pilot nature. Requests to access the datasets should be directed to Theresa Sukal-Moulton, theresa-moulton@northwestern.edu.

## Ethics Statement

The studies involving human participants were reviewed and approved by Northwestern University Institutional Review Board and Chicago Public School Research Review Board. Written informed consent to participate in this study was provided by the participants' legal guardian/next of kin. Written informed consent was obtained from the minor(s)' legal guardian/next of kin for the publication of any potentially identifiable images or data included in this article.

## Author Contributions

TS-M, TE, LJ, CL, and DG-S designed the program and study. TS-M, TE, LJ, and CL administered the program, collected data, and summarized results. TS-M, TE, and DG-S drafted the manuscript. All authors have reviewed and edited the manuscript. All authors contributed to the article and approved the submitted version.

## Funding

This work was supported by the American Academy of Cerebral Palsy and Developmental Medicine (AACPDM) Research Grant and the Spencer Foundation (Rapid Impact Grant to Northwestern University). REDCap was supported at FSM by the Northwestern University Clinical and Translational Science (NUCATS) Institute, Research reported in this publication was supported, in part, by the National Institutes of Health's National Center for Advancing Translational Sciences, Grant No. UL1TR001422.

## Conflict of Interest

The authors declare that the research was conducted in the absence of any commercial or financial relationships that could be construed as a potential conflict of interest.

## Publisher's Note

All claims expressed in this article are solely those of the authors and do not necessarily represent those of their affiliated organizations, or those of the publisher, the editors and the reviewers. Any product that may be evaluated in this article, or claim that may be made by its manufacturer, is not guaranteed or endorsed by the publisher.
